# Storax Attenuates Cardiac Fibrosis following Acute Myocardial Infarction in Rats via Suppression of AT1R–Ankrd1–P53 Signaling Pathway

**DOI:** 10.3390/ijms232113161

**Published:** 2022-10-29

**Authors:** Zhuo Xu, Danni Lu, Jianmei Yuan, Liying Wang, Jiajun Wang, Ziqin Lei, Si Liu, Junjie Wu, Jian Wang, Lihua Huang

**Affiliations:** 1 State Key Laboratory of Southwestern Chinese Medicine Resources, Chengdu University of Traditional Chinese Medicine, Chengdu 611137, China; 2 School of Pharmacy, Chengdu University of Traditional Chinese Medicine, Chengdu 611137, China

**Keywords:** storax, myocardial infarction, cardiac fibrosis, cardiac dysfunction, AT1R–Ankrd1–P53

## Abstract

Myocardial fibrosis following acute myocardial infarction (AMI) seriously affects the prognosis and survival rate of patients. This study explores the role and regulation mechanism of storax, a commonly used traditional Chinese medicine for treatment of cardiovascular diseases, on myocardial fibrosis and cardiac function. The AMI rat model was established by subcutaneous injection of Isoproterenol hydrochloride (ISO). Storax (0.1, 0.2, 0.4 g/kg) was administered by gavage once/d for 7 days. Electrocardiogram, echocardiography, hemodynamic and cardiac enzyme in AMI rats were measured. HE, Masson, immunofluorescence and TUNEL staining were used to observe the degree of pathological damage, fibrosis and cardiomyocyte apoptosis in myocardial tissue, respectively. Expression of AT1R, CARP and their downstream related apoptotic proteins were detected by WB. The results demonstrated that storax could significantly improve cardiac electrophysiology and function, decrease serum cardiac enzyme activity, reduce type I and III collagen contents to improve fibrosis and alleviate myocardial pathological damage and cardiomyocyte apoptosis. It also found that storax can significantly down-regulate expression of AT1R, Ankrd1, P53, P-p53 (ser 15), Bax and cleaved Caspase-3 and up-regulate expression of Mdm2 and Bcl-2. Taken together, these findings indicated that storax effectively protected cardiomyocytes against myocardial fibrosis and cardiac dysfunction by inhibiting the AT1R–Ankrd1–P53 signaling pathway.

## 1. Introduction

According to research data, cardiovascular disease is still one of the leading reasons for death worldwide, with approximately 17.9 million people dying from cardiovascular disease, accounting for 31% of worldwide deaths [1]. The number of deaths is estimated to increase to more than 23.6 million by 2030 [2]. Acute myocardial infarction (AMI), as a serious cardiovascular disease, is the main cause of cardiogenic shock, accounting for more than 80% [3]. In clinical practice, percutaneous transluminal angioplasty, thrombolysis and beta-blockers, angiotensin-converting enzyme inhibitors, aldosterone antagonists and angiotensin II (Ang II) receptor antagonists are mostly used. Direct percutaneous coronary intervention and reperfusion therapy with intravenous thrombolysis are used as the recommended treatment strategy for patients with acute ST-segment elevation myocardial infarction (STEMI) because they can save a dying myocardium and reduce the size of a myocardial infarction. However, even with early reperfusion therapy, STEMI patients still have a mortality rate of 10% within 1 year [4]. At the same time, the limited time window leaves some patients without restoration of effective perfusion after bypass or stent placement. Furthermore, ischemia/reperfusion-induced myocardial tissue injury also carries an increased risk of mortality and multiple complications in patients with myocardial infarction [5]. In addition, AMI always causes varying degrees of left ventricular remodeling, often decreasing coronary blood flow, exacerbating myocardial fibrosis and worsening cardiac function, which leaves patients with no radical improvement in clinical symptoms and prognosis [6]. Myocardial fibrosis post AMI is crucial for survival of patients. Nevertheless, most current Western therapeutic drugs act on a particular pathology of AMI, necessitating multi-drug combinations or integration of reperfusion therapy, as well as new medical device tools, which also have an increased risk of combined drug use [7]. Therefore, treatment of patients with myocardial infarction should not only focus on the occluded artery but should also be devoted to improving myocardial fibrosis and reducing ischemic necrosis, which has important scientific significance and research necessity.

According to the study, correct application of traditional Chinese medicine therapy while following modern medical treatment protocols can effectively improve the clinical symptoms and prognosis of patients with coronary heart disease (CHD) and enhance clinical outcomes [8]. Storax, the fragrant resin exuded from the trunk of *Liquidambar orientalis* Mill. of the Hamamelidaceae plant, has been refined into a semi-liquid thick liquid [9]. Its efficacy in treatment of CHD is definite, and it is widely used. Many countries have relevant medicinal records, and it is also included in the United States Pharmacopoeia [10]. There are hundreds of clinical prescriptions containing storax, such as Suhexiang pill and Guanxin Suhe pill, which are used for treatment of CHD with clear efficacy, low adverse effects and high patient compliance, in addition to having good application prospects [11]. Modern pharmacological studies have also shown that storax has pharmacological effects, such as anti-myocardial ischemia, anti-arrhythmia, anti-platelet aggregation, anti-inflammation and anti-apoptosis [12,13,14]. Yet, there are few reports on the effect and mechanism of storax on myocardial fibrosis after AMI.

Apoptosis plays an important role in cardiac insufficiency and myocardial structural changes after AMI and is involved in development of myocardial fibrosis and subsequent cardiac insufficiency until the appearance of symptomatic heart failure [15]. Therefore, anti-cardiomyocyte apoptosis is considered as an essential intervention in myocardial fibrosis as well as cardiac function. Accumulating evidence suggests that activation of the renin–angiotensin–aldosterone system (RAAS), which triggers apoptosis, is also a major pathogenic mechanism involved in myocardial fibrosis [16,17]. Hence, inhibition of the RAAS pathway, such as blocking the Ang Ⅱ type 1 receptor (AT1R), has proven to be an effective strategy for the current clinical treatment of myocardial fibrosis as well as for improving myocardial dysfunction [18]. It has been reported that ankyrin repeat domain 1 (Ankrd1), also known as cardiac ankyrin repeat protein (CARP), is a nuclear transcriptional cofactor accompanying Ang II activation and is involved in physiological and pathological remodeling of the myocardium [19,20]. Hypertrophic stimulation and heart failure rapidly induce Ankrd1 expression in cardiomyocytes [21]. It has also been shown that Ankrd1, a downstream molecule of AT1R, promotes heart failure by accelerating myocardial apoptosis through activation of the P53 mitochondrial apoptosis pathway [22,23]. These studies all indicated that Ankrd1 has a critical modulatory function in cardiovascular disease and is another attractive target for myocardial fibrosis. 

Nonetheless, there is no report on whether storax can effectively ameliorate myocardial fibrosis and cardiac diastolic dysfunction by modulating the AT1R/Ankrd1/P53 pathway to induce cardiomyocyte apoptosis. Consequently, this study is the first to explore the myocardial protective effect of storax by improving cardiac function and extracellular matrix remodeling, and this study may provide a new approach and strategy for the myocardial protective effect and mechanism of storax in treatment of coronary heart disease.

## 2. Results

### 2.1. Quantitative Analysis of Characteristic Components in Storax

**Figure 1:**
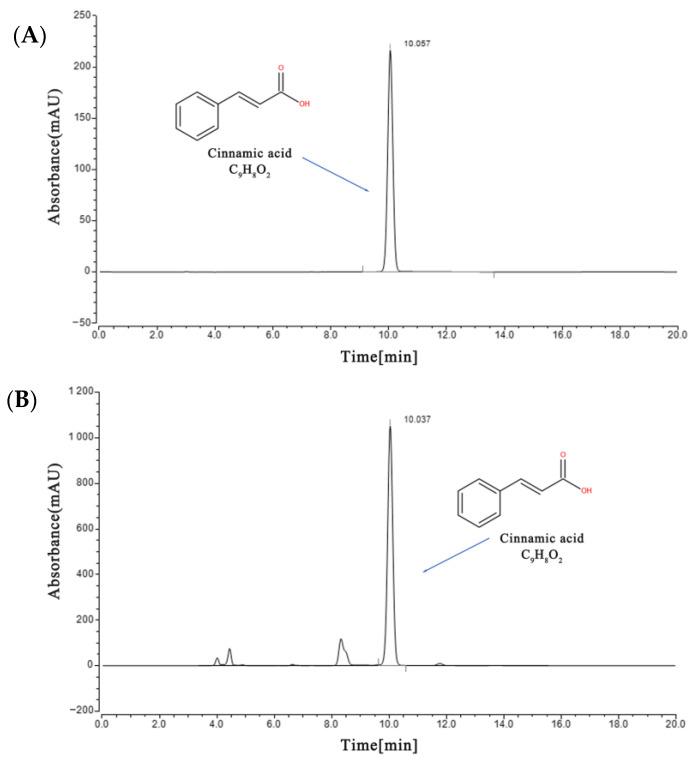
Quantitative analysis of representative chromatograms by HPLC. (**A**) Cinnamic acid (CA) reference substance. (**B**) Storax test substance.

Quantification of cinnamic acid (CA), the main component of storax, was performed by high performance liquid chromatography (HPLC), and the characteristic profile of CA was shown in Figure 1A and the characteristic profile of storax in Figure 1B. The standard curve was established according to the plotted peak area versus control concentration as y = 1.3946X − 0.1411, r^2^ = 0.9999 and the detection limit was 1–64 ug/mL. The content of CA in the test sample of storax oil was 79.38 mg/g by external standard method, which was greater than 5%, as stipulated in the Pharmacopoeia of the People’s Republic of China (2020 edition) [9], and the content was qualified.

### 2.2. Effect of Storax on Cardiac Electrophysiology and Cardiac Function in Isoproterenol Hydrochloride (ISO)-Induced AMI Rats

#### 2.2.1. Effect on the Electrocardiogram in AMI Rats

**Figure 2:**
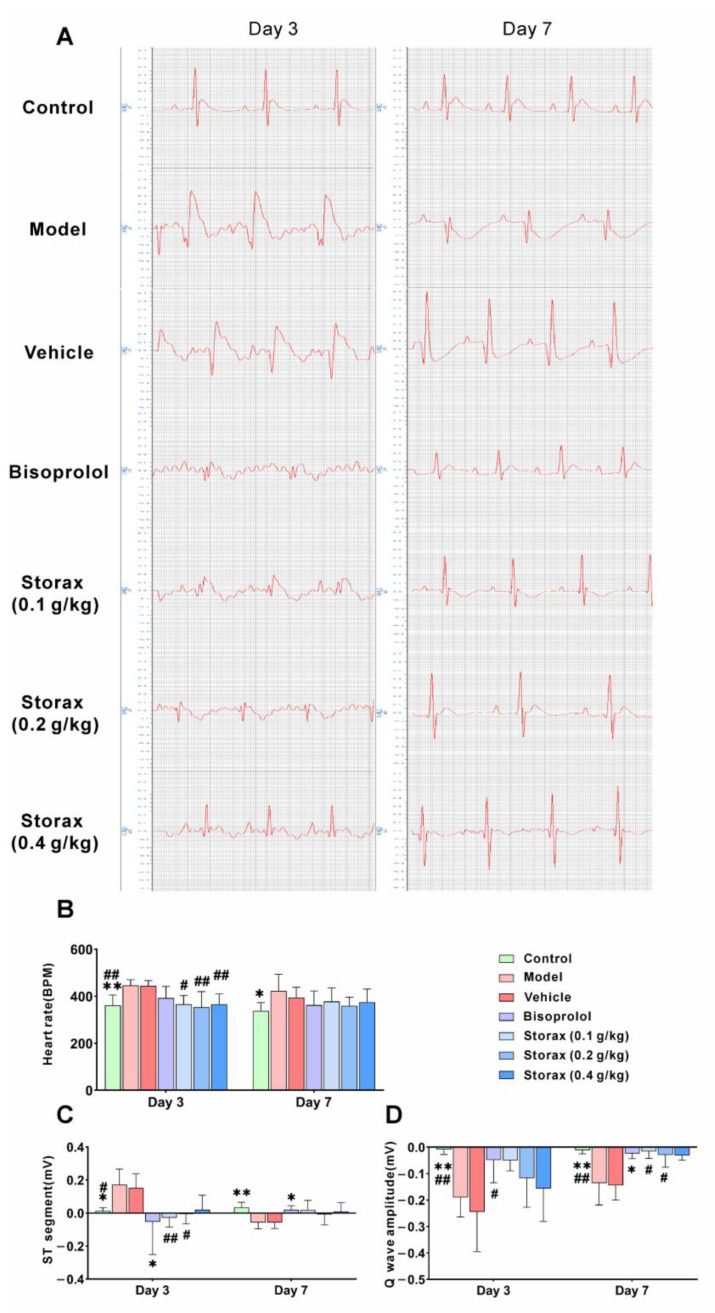
Effect of storax on the electrocardiogram of Isoproterenol hydrochloride (ISO)-induced acute myocardial infarction (AMI) rats. (**A**) Representative images of electrocardiogram. (**B**) Heart rate (HR). (**C**) ST-segment amplitude. (**D**) Q-wave amplitude. Results are presented as mean ± SD (n = 8). One-way ANOVA followed by Bonferroni’s post hoc test: * *p* < 0.05, ** *p* < 0.01 vs. model group; # *p* < 0.05, ## *p* < 0.01 vs. vehicle group.

Electrocardiograms were collected on day 3 and day 7. As shown in Figure 2A–D, on day 3, compared with the control group, the heart rate (HR) of the model group was significantly increased, the ST segment was significantly elevated and a deep Q-wave was formed, suggesting that the rats developed AMI after ISO injection. Compared with the model group, there was no significant difference in the physiological signal of the vehicle group, and the bisoprolol group could significantly reduce the heart rate, lower the ST-segment wave amplitude and inhibit the Q-wave deepening on day 7 and also significantly reduce the ST-segment wave amplitude on day 3. Compared with the vehicle group, all three dose groups of storax significantly slowed down the heart rate, reduced the ST-segment amplitude and inhibited the Q-wave deepening in rats on day 3. On day 7, the storax 0.1 and 0.2 g/kg groups could suppress Q-wave deepening. 

#### 2.2.2. Effect on Cardiac Function in AMI Rats 

**Figure 3:**
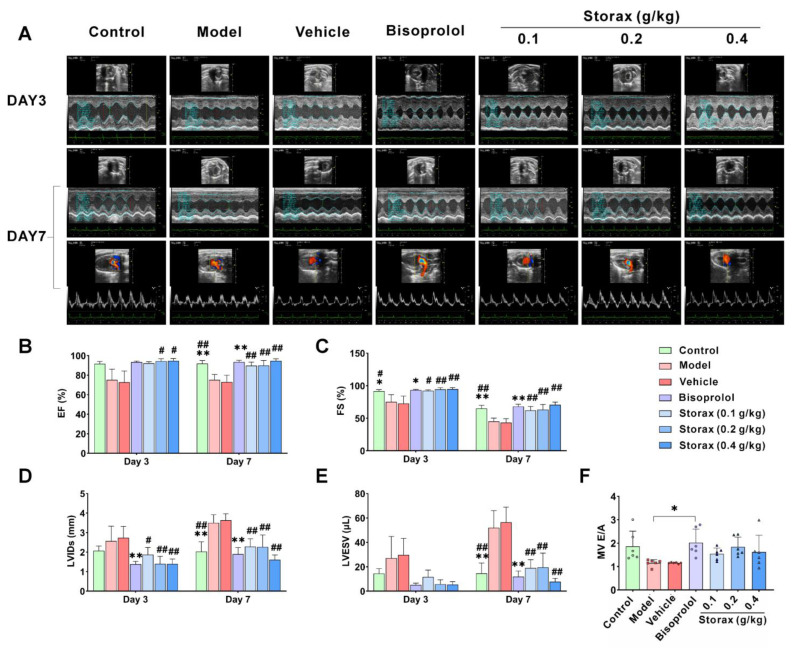
Effect of storax on cardiac function in ISO-induced AMI rats. (**A**) Representative images of M-mode echocardiography and mitral pulse Doppler. (**B**) Ejection fraction (EF). (**C**) Shortening fraction (FS). (**D**) Left ventricular end-systolic inner diameters (LVIDs). (**E**) Left ventricular end-systolic volume (LVESV). (**F**) MV E/A. Results are presented as mean ± SD (n = 6). One-way ANOVA followed by Bonferroni’s post hoc test: * *p* < 0.05, ** *p* < 0.01 vs. model group; # *p* < 0.05, ## *p* < 0.01 vs. vehicle group.

To investigate the protective effect of storax on cardiac function in rats, a small animal ultrasound system was used to dynamically monitor cardiac function. As shown in Figure 3A–E, on day 7, compared with the control group, both ejection fraction (EF) and shortening fraction (FS) were significantly decreased, and left ventricular end-systolic inner diameters (LVIDs) and left ventricular end-systolic volume (LVESV) were significantly increased in the model group. There were no significant differences in all the cardiac function parameters between the vehicle group and the model group. The bisoprolol group significantly increased EF and FS and significantly decreased LVIDs and LVESV. Compared with the vehicle group, the storax 0.2 and 0.4 g/kg groups could significantly increase EF and FS on day 3 and day 7 and significantly reduce LVIDs and LVESV, with a trend of increasing the ratio of the E peak (E) to A peak (A) (Figure 3F). 

**Figure 4:**
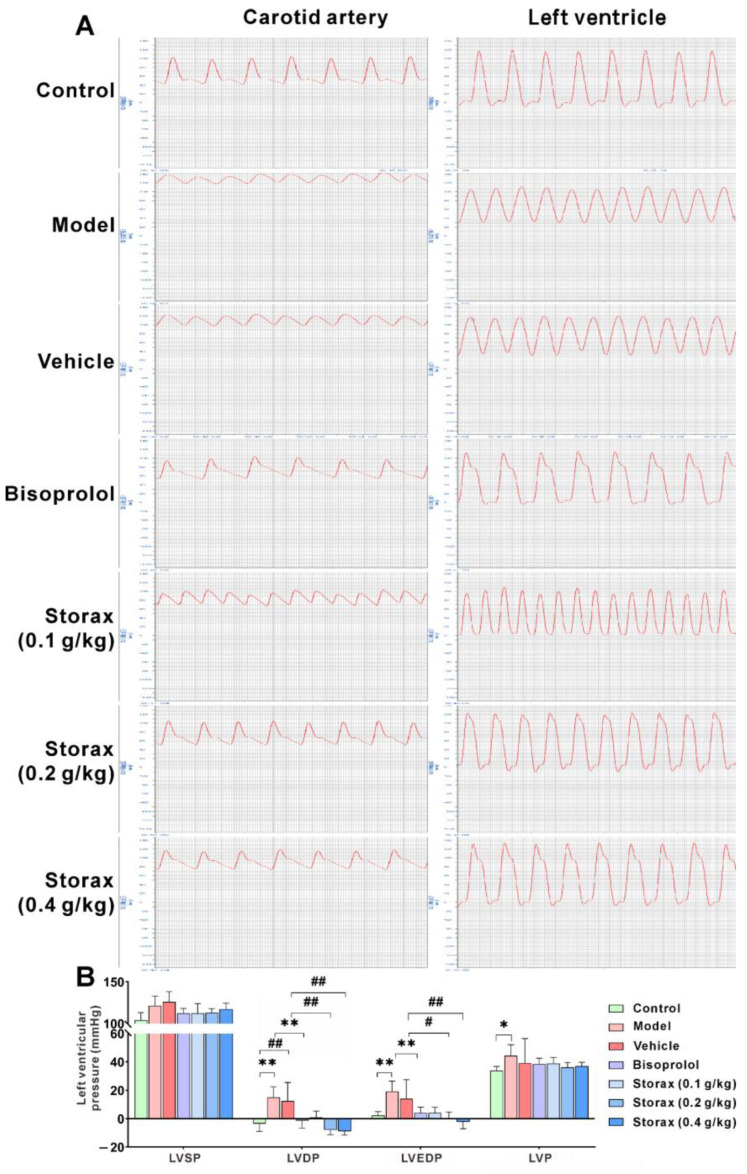

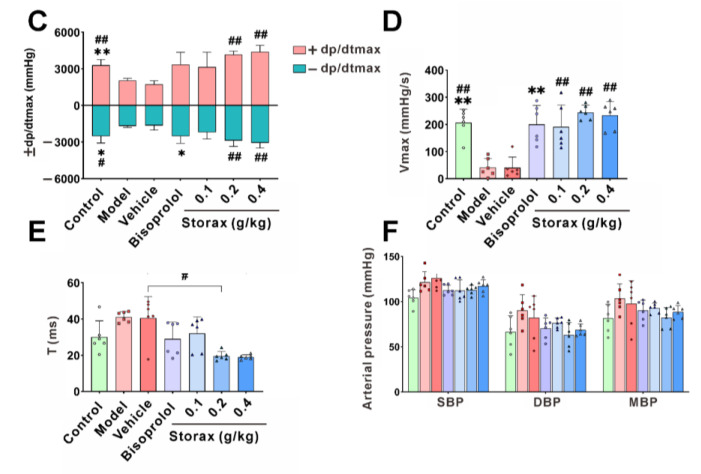
Effect of storax on hemodynamics in ISO-induced AMI rats. (**A**) Representative images of hemodynamics. (**B**) Quantitative analysis of left ventricular pressure: left ventricular systolic blood pressure (LVSP), left ventricular diastolic pressure (LVDP), left ventricular end-diastolic pressure (LVEDP), left ventricular pressure (LVP). (**C**) Quantitative analysis of ±dp/dtmax. (**D**) Quantitative analysis of left ventricular myocardial contractile component measured maximum shortening velocity (Vmax). (**E**) Quantitative analysis of left ventricular pressure drop time (T). (**F**) Quantitative analysis of arterial pressure: mean arterial pressure (MAP), systolic blood pressure (SBP), diastolic blood pressure (DBP). Results are presented as mean ± SD (n = 6). One-way ANOVA followed by Bonferroni’s post hoc test: * *p* < 0.05, ** *p* < 0.01 vs. model group; # *p* < 0.05, ## *p* < 0.01 vs. vehicle group.

To further evaluate and verify the blood supply function of the heart, an invasive catheter insertion was used to determine the cardiac hemodynamics in rats. Compared with the control group, arterial pressure left ventricular diastolic pressure (LVDP), left ventricular end-diastolic pressure (LVEDP), the maximum falling velocity of left ventricular pressure (−dp /dt max) and left ventricular pressure drop time (T) were significantly higher in the model group, and left ventricular systolic force, maximum rising velocity of left ventricular pressure (+dp/dt max) and left ventricular myocardial contractile component measured maximum shortening velocity (Vmax) were significantly lower. Compared with the model group, there was no significant difference in the vehicle group. The bisoprolol group and the storax 0.2 and 0.4 g/kg groups reduced LVDP, LVEDP, −dp/dtmax and T to varying degrees, and enhanced +dp/dtmax and Vmax (Figure 4A–E). There was a trend of elevated arterial blood pressure after modeling, and both bisoprolol and storax treatment had a tendency to lower arterial blood pressure (Figure 4F). 

### 2.3. Storax Improved Myocardial Injury and Fibrosis in ISO-Induced AMI Rats

**Figure 5:**
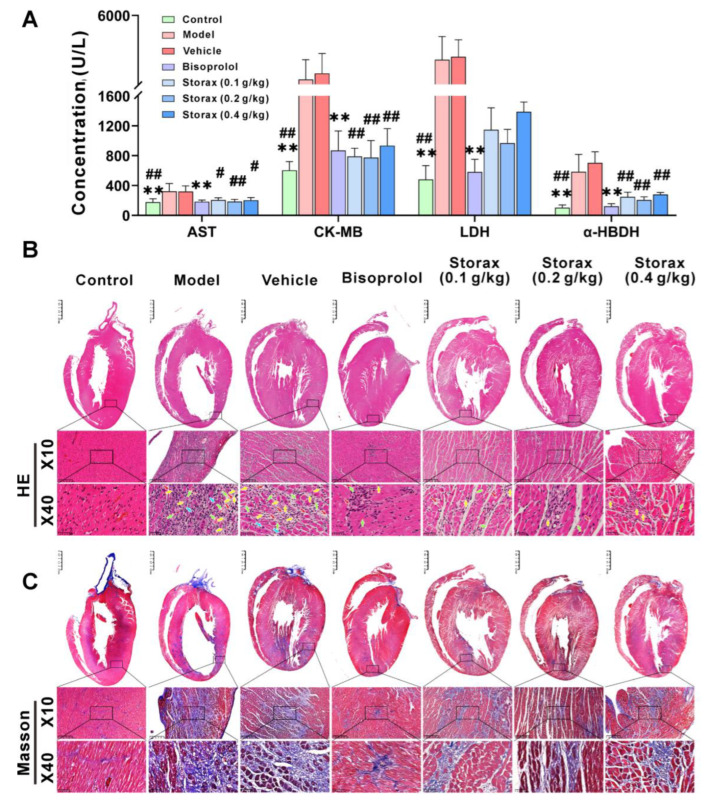

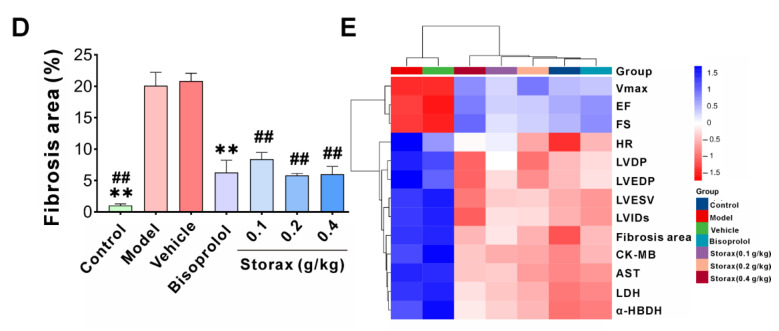
Effect of storax on myocardial injury and fibrosis in ISO-induced AMI rats. (**A**) Quantitative analysis of the expression levels of aspartate aminotransferase (AST), creatine kinase iso-enzyme (CK-MB), lactate dehydrogenase (LDH) and α-hydroxybutyrate dehydrogenase (α-HBDH) in serum of rats in different groups (n = 6). (**B**) Representative images of hematoxylin–eosin (HE) staining in each group (scale bar = 250 μm or 50 μm, n = 4), vascular congestion (green arrows), macrophages (blue arrows), edema (red arrows), necrosis (yellow arrows). (**C**) Representative images of Masson staining in each group (scale bar = 250 μm or 50 μm, n = 4). (**D**) Quantitative analysis of the proportion of collagen fibers. The blue-stained area is collagen fiber organization. (**E**) Heat map of cluster analysis of biochemical indexes regulated by different groups. Results are presented as mean ± SD. One-way ANOVA followed by Bonferroni’s post hoc test: ** *p* < 0.01 vs. model group; # *p* < 0.05, ## *p* < 0.01 vs. vehicle group.

To determine whether storax has a protective effect on ISO-induced myocardial injury, the levels of myocardial injury biomarkers, such as lactate dehydrogenase (LDH), aspartate aminotransferase (AST), creatine kinase isoenzyme (CK-MB) and α-hydroxybutyrate dehydrogenase (α-HBDH), in rat serum were measured, thereby reflecting the severity of myocardial injury. As shown in Figure 5A, compared with the control group, serum AST, LDH, CK-MB and α-HBDH were significantly increased in the model group. Compared with the model group, there was no significant difference in the vehicle group, while the bisoprolol group could significantly reduce the serum levels of related markers. Compared with the vehicle group, each dose group of storax could significantly inhibit serum biomarkers.

In order to confirm the effect of storax on the histopathology of myocardial injury and myocardial fibrosis, hematoxylin–eosin (HE) staining and Masson staining were separately carried out. The results of HE staining are shown in Figure 5B. In the control group, the structure of the cardiac tissue was neatly and regularly arranged, the nuclei were intact and evenly stained and the size of the cardiomyocytes was normal with clear boundaries. The model group showed thickened myocardial fibers, disorganized arrangement, enlarged intercellular spaces and a large number of vascular congestion, cell edema and focal necrosis near the myocardial fibers. The vehicle group was similar to the model group. Myocardial fiber thickening and edema were significantly improved in the storax 0.1, 0.2, 0.4 g/kg dose groups and bisoprolol group. In these groups, the arrangement of cells was more regular, the structure of cardiomyocytes was more complete and the infiltration of inflammatory cells was significantly reduced. Among them, the storax 0.2 g/kg group had the most obvious effect, which was comparable to the bisoprolol group.

Masson staining was used to investigate the changes in collagen content of myocardial tissue; the blue area was collagen fibrous tissue and the red area was cardiomyocytes, fibroblasts and erythrocytes; the results are shown in Figure 5C,D. Compared with the control group, a large blue-stained area was observed in the myocardial tissue of rats in the model group, which showed proliferation of reticular fibrous connective tissue, suggesting that the myocardial tissue of rats in the model group showed obvious fibrosis. The vehicle group was similar to the model group. The blue-stained area in the myocardial tissue of rats in the three dose groups of storax as well as the bisoprolol group was significantly reduced.

These above results suggest that both storax and bisoprolol treatment can effectively mitigate ISO-induced myocardial injury and myocardial fibrosis. The important efficacy indexes were normalized by Z-score and then clustered for analysis. As shown in Figure 5E, the comprehensive evidence suggests that the storax 0.2 g/kg group is more effective, so it provides a basis for setting of groups for subsequent mechanism research.

### 2.4. Storax Inhibited the Expression of Type I and III Collagen in ISO-Induced AMI Rats

**Figure 6:**
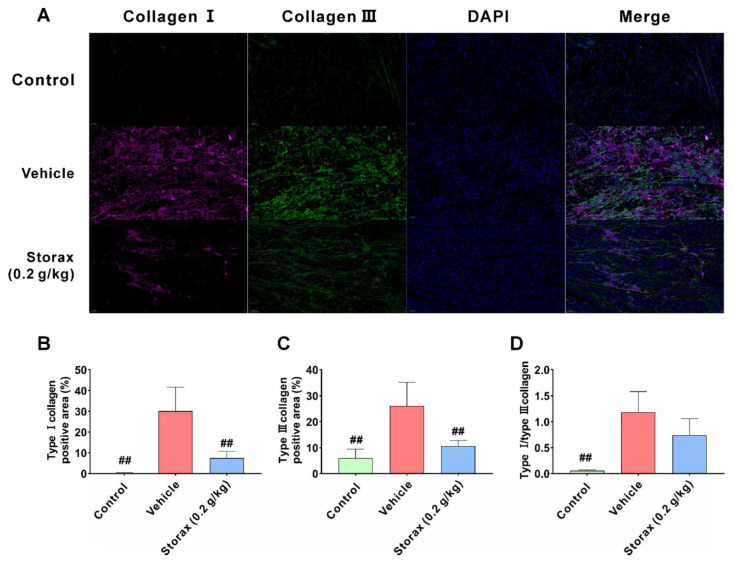
The effect of storax on the expression of type I and III collagen in the myocardium of ISO-induced AMI rats. (**A**) Representative images of myocardial type I and III collagen immunofluorescence staining in each group of rats. Type I collagen immunofluorescence staining was shown in pink, type III collagen immunofluorescence staining was shown in green and DAPI nuclear staining was shown in blue (scale bar = 50 μm). (**B**) Quantitative analysis of collagen type I. (**C**) Quantitative analysis of collagen type III. (**D**) Quantitative analysis of the ratio of collagen I/III. Results are presented as mean ± SD (n = 4). One-way ANOVA followed by Bonferroni’s post hoc test: ## *p* < 0.01 vs. vehicle group.

To elucidate the mechanism of storax on myocardial fibrosis, immunohistofluorescence was used to detect the expression of type I and III collagen in myocardial tissue. Compared with the control group, the deposition of type I and collagen in the vehicle group increased significantly, and the ratio of type I collagen to type III collagen also increased, suggesting that type I collagen synthesis was more obvious than type III collagen. Compared with the vehicle group, the storax 0.2 g/kg group could significantly reduce expression of type I and type III collagen, and there was a tendency to decrease the ratio of type I to III collagen (Figure 6A,B).

### 2.5. Storax Inhibited Cardiomyocyte Apoptosis in ISO-Induced AMI Rats

**Figure 7:**
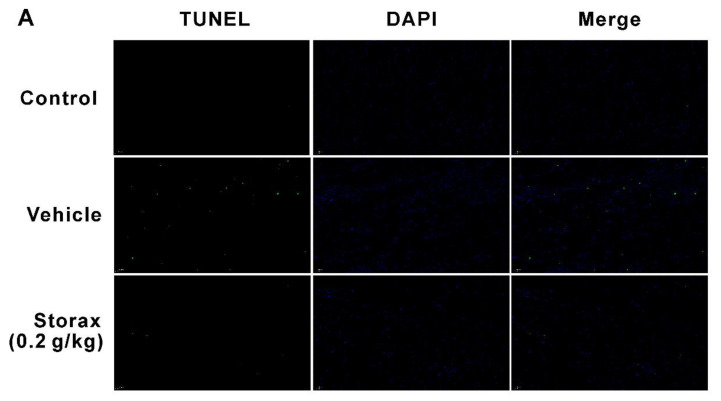

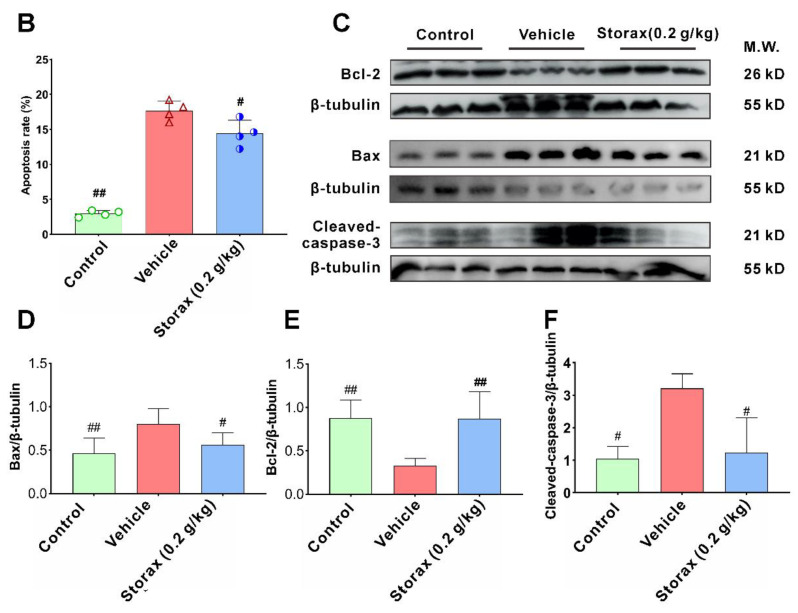
Effects of storax on cardiomyocyte apoptosis in the myocardium of ISO-induced AMI rats. (**A**) Representative images of terminal deoxynucleotidyl-transferase-mediated dUTP nick end labeling (TUNEL) staining. TUNEL-positive apoptotic cells were shown in green and DAPI nuclear staining was shown in blue (scale bar = 50 μm). (**B**) Quantitative analysis of TUNEL-positive cells (n = 4). (**C**) The protein expression levels of Bax, Bcl-2 and cleaved Caspase-3 in rat cardiac tissue of each group were shown by Western blotting (WB) bands. (**D**–**F**) Relative protein for Bax, Bcl-2 and cleaved Caspase-3 were quantified by densitometry based on immunoblot images. Results are presented as mean ± SD (n = 3). One-way ANOVA followed by Bonferroni’s post hoc test: # *p* < 0.05, ## *p* < 0.01 vs. vehicle group.

The results of terminal deoxynucleotidyl-transferase-mediated dUTP nick end labeling (TUNEL) staining were shown in Figure 7A,B. Few TUNEL-positive cells were observed in the control group. Compared with the control group, the number of TUNEL-positive cells in the vehicle group increased significantly. Compared with the vehicle group, the storax 0.2 g/kg group could significantly reduce the number of TUNEL-positive cells in heart tissue. It was suggested that storax could attenuate cardiomyocyte apoptosis in ISO-induced post-infarction myocardial fibrosis. To confirm the anti-apoptotic effect of storax, mitochondrial-apoptosis-related proteins, such as Bax, Bcl-2 and cleaved Caspase-3 in myocardial tissues, were detected by Western blotting (WB). The results are shown in Figure 7C–F. Compared with the control group, Bax and cleaved Caspase-3 were significantly increased and Bcl-2 was decreased in the vehicle group. Storax significantly down-regulated expression of Bax and cleaved Caspase-3 and significantly increased expression of Bcl-2. 

### 2.6. Storax Reduced ISO-Induced Cardiomyocyte Apoptosis in AMI Rats by Inhibiting AT1R– Ankrd1–P53 Pathway

**Figure 8:**
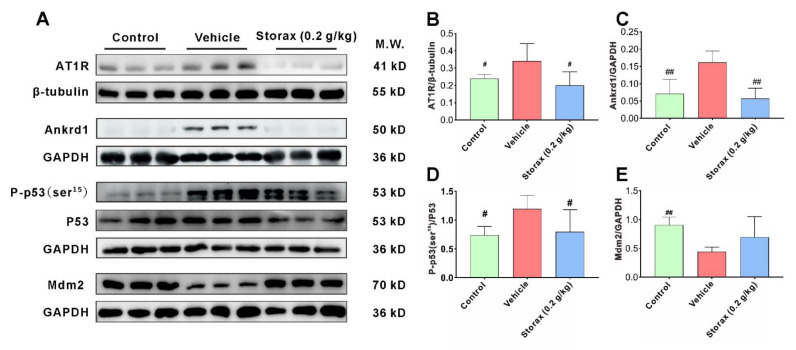
Storax inhibits the AT1R–Ankrd1–P53 pathway in AMI rats against cardiomyocyte apoptosis. (**A**) WB bands showing the protein expression levels of AT1R, Ankrd1, P-p53 (ser15), P53 and Mdm2 in cardiac tissue. (**B**–**E**) Relative protein for AT1R, Ankrd1, P-p53 (ser15) and Mdm2 were quantified by densitometry based on immunoblot images. Results are presented as mean ± SD (n = 3). One-way ANOVA follow by Bonferroni’s post hoc test: # *p* < 0.05, ## *p* < 0.01 vs. vehicle group.

In order to illustrate the mechanism of storax against cardiomyocyte apoptosis, the expression levels of AT1R, Ankrd1, P53, P-p53 (ser15) and Mdm2 in myocardial tissue were examined by WB (Figure 8A). Compared with the control group, the expression levels of AT1R, Ankrd1 and P-p53 (ser15) in myocardial tissue of vehicle group rats were significantly increased, and the expression level of Mdm2 was significantly decreased. Storax significantly down-regulated the expression levels of AT1R, Ankrd1 and P-p53 (ser15) and increased the expression level of Mdm2.

## 3. Discussion

AMI is one of the acute coronary syndromes with high mortality and morbidity worldwide [24]. Myocardial infarction occurs when an ischemic state occurs following myocardial cell hypoxia, leading to irreversible myocardial damage. ISO is a β-adrenergic receptor agonist. A large dose of ISO can cause severe myocardial contraction, resulting in coronary spasm, myocardial tissue ischemia and hypoxia, and can induce insufficient blood supply to myocardial cells, resulting in AMI. Nowadays, high-dose injection of ISO has become a common treatment method to induce AMI in rat models. Moreover, the ISO-mediated rat AMI model can better simulate the coronary spasm process at the onset of human AMI and is similar to the morphological and pathophysiological characteristics of human AMI [25]. Therefore, this experiment used ISO to establish the AMI rat model. Due to enlargement and dilatation of the left ventricle after AMI, the myocardium is unable to pump oxygenated blood, thus depriving the heart tissue of blood and leading to myocardial necrosis, followed by myocardial fibrosis in which the necrotic cells are replaced by collagen [26]. Cardiac fibrosis is characterized by thickening or scarring of heart valves due to inappropriate proliferation and aggregation of cardiac fibroblasts, resulting in distortion and dysfunction of heart shape and mechanical function [27]. These fibroblasts synthesize type I and type III fibrillar collagen, which are the main components of the myocardial interstitium. Type I collagen mainly forms coarse fibers, which are weakly stiff in extension and resilience. Type III collagen mainly forms fine meshwork, which expands more easily than type I collagen [28]. The appropriate ratio of type I and III collagen is valuable for maintaining the structure and function of the interstitial matrix. An increase in the total myocardial collagen content or an increase in the proportion of type I and III collagen will lead to disruption of the normal myocardial fiber network structure, limiting normal expansion and contraction of the myocardial cells, which will result in decreasing myocardial compliance and ejection capacity, increasing myocardial stiffness, impairing myocardial diastolic or systolic function and ultimately heart failure [29]. Hence, delaying or preventing myocardial fibrosis is a key component in the prevention and treatment of ventricular remodeling and heart failure after AMI. 

At present, for treatment of myocardial fibrosis after AMI, in addition to reconstructing the blood supply and limiting the infarct extent, early pharmacological intervention and treatment are still the main means. Storax, as a traditional Chinese medicine, is widely used in treatment of cardiovascular diseases, with modern pharmacological effects of anti-myocardial ischemia, improving blood rheology as well as hemodynamics [12,13]. CA, as an index component for the content determination of storax in the Pharmacopoeia of the People’s Republic of China (2020 edition), may also be one of the main components for its pharmacological effects. It has been demonstrated that CA has a cardioprotective effect on rats with myocardial ischemia-reperfusion injury [30]. However, the lack of in-depth and systematic mechanistic research has limited the application scope of storax and its constituent formulations.

In this study, the ST segment of ECG lead II of the rats after ISO injection was significantly higher than that of the control group, and a pathological Q-wave was formed, indicating that the AMI rat model was successfully established. Both EF and FS are common indicators of left ventricular (LV) systolic function, and their reduced values often indicate decreased myocardial contractility. LVIDs and LVESV are quantitative indicators of LV shape, and their values are closely related to ventricular remodeling [31,32,33]. The echocardiographic results showed that storax significantly increased EF and FS to enhance myocardial contractility and reduced LVIDs and LVESV to improve ventricular structure. The color Doppler ultrasound results also showed that storax can improve myocardial contractility in AMI rats and maintain the normal shape of the myocardium and inhibit left ventricular remodeling. Combined with the results of hemodynamics by inserting an arterial catheter, the 0.2 and 0.4 g/kg storax groups both reduced LVDP and LVEDP, −dp/dtmax and T to reduce left ventricular load, improve diastolic dysfunction and regulate ventricular diastolic function [34]. It also increases +dp/dtmax as well as Vmax to enhance myocardial contractility and facilitate enhanced cardiac pumping [35]. Based on the different significance of change in each parameter, this study evaluated the effect of storax in the treatment of AMI from a comprehensive and multifaceted perspective. Comprehensive tips include that storax has the effect of improving myocardial diastolic dysfunction and maintaining myocardial morphology and pumping function.

The expression levels of AST, LDH, CK-MB and α-HBDH in rat serum are biochemical indicators to detect the extent of cardiac injury [36]. The results of this study showed that the serum levels of AST, LDH, CK-MB and α-HBDH were significantly higher in the rats after ISO modeling than the control group, while the serum levels of the relevant biomarkers were significantly reduced after storax intervention. The combination of HE and Masson staining of cardiac histopathological results showed that storax had the effect of ameliorating myocardial cell edema, cell necrosis, vascular congestion, inflammatory cell infiltration, collagen deposition and down-regulation of fibrosis area caused by ISO, which again confirmed that storax was beneficial for repairing ISO-induced myocardial injury in AMI rats and had a positive effect on improving myocardial fibrosis. To facilitate an in-depth exploration of the myocardial protective mechanism of storax, the above indicators were combined for clustering analysis to extract a valid data structure. By observing the characteristics of each cluster obtained by clustering, further analysis can be focused on specific certain clusters [37]. The analysis showed that each administration group and the control group could be clustered into one category, proving the efficacy of the drug. Among them, the storax 0.2 g/kg group was the closest to the control group. Therefore, the storax 0.2 g/kg group was used as the subject for the subsequent mechanism study. The vehicle group and the model group were clustered into a separate category, and the above indicators were not significantly different between the model group as well as the vehicle group, indicating that the vehicle (2% Tween) did not significantly affect the results of the study. Therefore, only the vehicle group was set in the follow-up studies.

Continuous blood flow pressure overload leads to abnormal extracellular matrix metabolism, resulting in a change in the ratio of type I and III collagen [38]. The hemodynamic data showed that storax reduced blood pressure in AMI rats. Masson staining showed that the collagen blue staining area increased and the fibrosis area increased significantly, all of which indicated excessive deposition of collagen and formation of myocardial fibrosis after myocardial infarction. Immunofluorescence staining showed that storax could significantly reduce the deposition of type I and type III collagen in AMI rats and down-regulate the ratio of type I and type III collagen, thus improving the degree of myocardial fibrosis, which again verified the effect of storax in improving myocardial fibrosis in AMI rats.

There is increasing evidence that apoptosis plays an important role in myocardial infarction and myocardial fibrosis. Apoptosis in AMI can significantly induce cell death, leading to a decrease in the number of cardiomyocytes and progression of myocardial interstitial fibrosis, contributing to the incidence of myocardial remodeling. Thus, apoptosis is closely related to myocardial fibrosis [39]. After AMI, patients with symptomatic heart failure were associated with a significant increase in apoptosis [40,41]. Thereby, inhibition of cardiomyocyte apoptosis has become a research direction for treatment of myocardial fibrosis and heart failure. While the molecular mechanisms involved in apoptosis are complex, most scholars believe that the Bcl-2 family, cysteaspase (caspase) and other families are involved in the initiation, occurrence and development of apoptosis [42]. Pro-apoptotic protein Bax and apoptosis-inhibiting protein Bcl-2 restrict each other, and the ratio of the two reflects the state of apoptosis [43]. The caspase family is synthesized in the form of inactive precursor enzymes. Activated caspase molecule (cleaved Caspase-3) plays an important role in the cardiomyocyte apoptosis pathway, gradually disrupting cellular components through cleavage of specific substrates, reconstructing the cytoskeleton and degrading the nucleus. Among the members of the caspase family, caspase-3 is a key regulator of apoptosis. As a hub of endogenous and exogenous apoptotic pathways, caspase-3 plays an important role in the final stage of apoptosis [44]. Previous research found that storax can significantly reduce the ratio of Bax to Bcl-2 and the number of TUNEL-positive apoptotic cells, suggesting that storax can improve myocardial ischemia in rats induced by left anterior descending coronary artery ligation though inhibiting myocardial cell apoptosis [45]. This study also found that storax can reduce TUNEL-positive cells and down-regulated the ratio of Bax/Bcl and cleaved Caspase-3 in rats with myocardial fibrosis after ISO-induced AMI, which is consistent with the previous results. It suggested that storax may improve myocardial fibrosis in rats with AMI through the anti-apoptotic pathway. 

**Figure 9:**
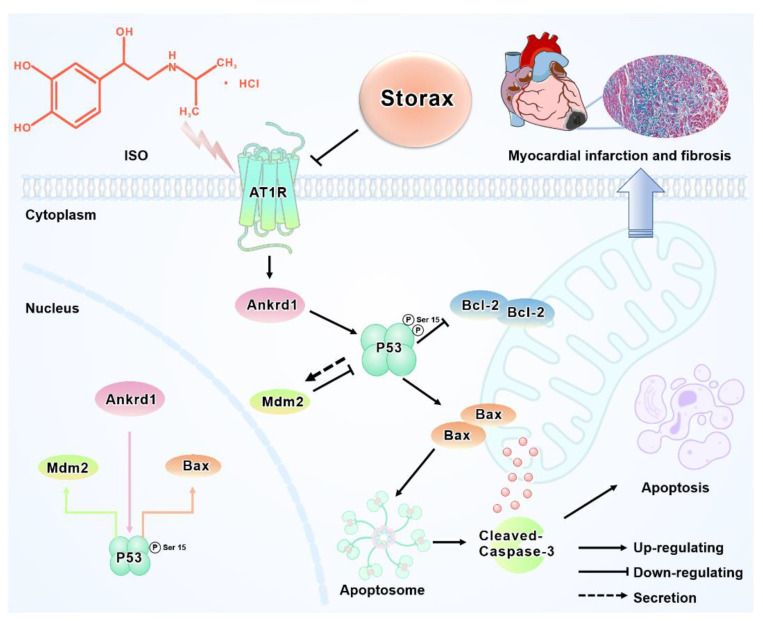
The underlying mechanisms of the cardioprotective activity of storax against ISO-induced AMI in rats. ISO-induced myocardial infarction can activate the AT1R–Ankrd1–P53 signaling pathway, thereby inducing cardiomyocyte apoptosis. Storax intervention effectively inhibited the AT1R–Ankrd1–P53 signaling pathway to suppress cardiomyocyte apoptosis and improve post-infarction myocardial fibrosis.

It has been demonstrated that changes in vascular pressure can lead to activation of the Ang Ⅱ-AT1R pathway, which, in turn, induces apoptosis in cardiac myocytes. ISO-induced myocardial ischemia-hypoxic injury activates the release of the neurohormone system RAAS and its effector AT1R, which play a crucial role in the process of ventricular remodeling after AMI [46]. Increased AT1R induces cardiomyocyte apoptosis [47]. Ankrd1, a cardiac-anchored repeat protein expressed post-transcriptionally, was found in the nucleus early as a transcriptional cofactor for cardiac gene expression [20]. It has been reported in the literature that Ankrd1 can be regulated by Ang II through its type 1 receptor AT1R and directly participate in the process of cardiomyocyte apoptosis and hypertrophy and has become a new potential target for treatment of heart failure [48]. Therefore, the AT1R–Ankrd1 pathway may be a pivotal pathway for apoptosis after myocardial infarction. The essence of apoptosis is DNA fragmentation caused by activation of endogenous endonucleases, and the process is associated with abnormal expression of various genes (such as P53, Bcl-2, etc.). Apoptotic gene P53 is a tumor suppressor gene. In recent years, it has been found that P53 can participate in the apoptotic process of cardiomyocytes through a negative regulatory effect on cell growth. It has also been found that Ankrd1 can participate in apoptosis by activating P53 [22]. Ankrd1 overexpression enhances cardiomyocyte apoptosis by promoting P53 activation and mitochondrial dysfunction [23]. As a ubiquitin ligase, MDM2 gene can effectively bind and ubiquitinate P53 and induce proteasomal degradation. It is a negative regulator of P53 and can degrade expression of P53 [49,50]. In this study, the WB method was used to investigate AT1R–Ankrd1–P53-regulated apoptosis, and the results showed that storax significantly down-regulated expression of AT1R as well as Ankrd1, inhibited expression of P53 and phosphorylated p53 and activated the expression of Mdm2, suggesting that the anti-apoptotic effect of storax may be exerted through the AT1R–Ankrd1–P53 pathway. In summary, this study explains the mechanism of action of storax against cardiomyocyte apoptosis in a more systematic way, and the integration results are shown in Figure 9, which provides valuable evidence to elucidate anti-myocardial fibrosis and the improvement in cardiac function of this drug. 

In this paper, we focus on the role of storax in improving myocardial fibrosis and cardiac dysfunction based on the overall level; however, in-depth elucidation of the biological mechanism of storax on cardiac fibroblasts based on the proteomics-cellular level is the next scientific question to be addressed by the author. Subsequently, the active components will also be screened out, and multi-omics and multiple research tools will be combined to predict the targets of action of the active substances, which will provide a reference for elucidation of the mechanism and expansion of its application.

## 4. Materials and Methods

### 4.1. Experimental Drugs and Reagents

CA (purity ≥ 98%, CAS: 140-10-3) was purchased from Chengdu Chroma-Biotechnology Co., Ltd. (Chengdu, China). Refined storax oil (origin was Turkey) was purchased from the Chinese herbal medicine market (Chengdu, China). Bisoprolol fumarate tablets were purchased from Beijing Huasu Pharmaceutical Co., Ltd. (Beijing, China). Isoproterenol hydrochloride (ISO) (purity ≥ 98%, CAS: 51-30-9) was purchased from Shanghai Rhawn Co., Ltd. (Shanghai, China). Tween-80 (lot: 2019070301), urethane (lot: 2020041701) and formaldehyde solution (lot: 180417) were purchased from Chengdu Chron Chemicals Co., Ltd. Company (Chengdu, China). Isoflurane (lot: 21071801) was purchased from RWD Life Science Co., Ltd. (Shenzhen, China). LDH (lot: 142721004), AST (lot: 140221004), CK-MB (lot: 142621002) and α-HBDH (lot: 140521003) kits were purchased from Shenzhen Mindray Biomedical Electronics Co., Ltd. Co., Ltd. (Shenzhen, China). TYR-690, RCF021 and TYR-520 were purchased from Shanghai Record Biology Technology Co., Ltd. (Shanghai, China). UltraSignal ECL WB Detection Reagent (product number: 4AW011-100) was purchased from 4A Biotech.,Ltd. (Beijing, China). DAPI, TUNEL staining kit, RIPA lysis solution, BCA kit and Stripping buffer were purchased from Servicebio. Co., Ltd. (Wuhan, China).

### 4.2. Chemical Profile Analysis of Storax

The Pharmacopoeia of the People’s Republic of China (2020 edition) stipulates that CA contained in storax should not be less than 5%, so the content of CA in storax was determined by HPLC with reference to the pharmacopoeia [9]. The storax oil sample was weighed precisely 0.5 g in a 50 mL brown volumetric flask. The volume was fixed to the scale with methanol, then dissolved by ultrasonication for 20 min. Add 2 mL into a 10 mL brown volumetric flask, add methanol and fix the volume to the scale, vortex and mix to obtain 2 mg/mL sample. CA standard (10 mg) was ultrasonicated in 10 mL methol to make a solution of 1 mg/mL, and aspirate 100 μL to 900 μL methanol to make a solution of 100 μg/mL. Aspirate 640 μL into 360 μL methanol to make 64 μg/mL solution, dilute in gradient and prepare 32, 16, 8, 4, 2, 1 μg/mL solution, respectively. The samples were filtered through 0.22 μm microporous membranes before injection. 

Further, 10 μL of the supernatants was injected into a Thermo HPLC system equipped with an online degasser, a quaternary solvent delivery system, an autosampler and a diode-array detector (285 nm). An analytical Acquity C18 chromatographic column (3.0 × 100 mm,1.8 μm) with a flow rate of 1.0 mL/min and a column temperature of 30 °C was used for the analysis. The mobile phase consisted of methanol and 1% glacial acetic acid solution (50:50). The retention times (RT) and area of the peak of the storax sample were compared to those measured in the standard samples to identify and determine the content of CA in the storax sample. 

### 4.3. Animals and Experimental Design

Male Sprague Dawley (SD) rats (weighing 250 ± 20 g, 8–10 weeks old) were obtained from SPF (Beijing) Biotechnology Co., Ltd. (Beijing, China), production license number: SCXK (Jing) 2019-0010. The animals drank water and dieted freely with 22 ± 2 °C room temperature. The humidity was 40–70% with a 12 h light/12 h dark cycle. All experimental procedures and protocols were conducted strictly in accordance with the Administrative Regulations on Laboratory Animals formulated by China and were approved by the Laboratory Animal Ethics Committee of Chengdu University of Traditional Chinese Medicine (No.: 2019DL-002).

The animals were randomly divided into control group, model group, vehicle group, bisoprolol group (positive control), storax 0.1, 0.2 and 0.4 g/kg groups, a total of 7 groups. Except for the control group, the other groups were injected subcutaneously with ISO dissolved in saline to induce AMI, 75 mg·kg^−1^·d^−1^, 24 h apart, for 2 d. The storax oil was added dropwise to the 2% Tween-80 solution, emulsified by continuous grinding and diluted with physiological saline to the desired concentrations: 0.01, 0.02, 0.04 g/mL storax. The dosages of ISO and storax were chosen based on preliminary experiments and previous literature [51,52]. 

All rats were gavaged at 10 mL/kg and grouped as follows:

(1) Control group: equal volume of saline was given to all in the same administration;
(2) Model group: ISO (sc) + equal volume of saline (i.g);
(3) Vehicle group: ISO (sc) + equivalent 2% Tween solvent (i.g);
(4) Bisoprolol group: ISO (sc) + 2.5 mg/kg bisoprolol (i.g, dissolved in saline, at a clinically equivalent dose of 2.5 mg/kg/d);
(5) 0.1 g/kg storax group: ISO (sc) + 0.1 g/kg/d storax (i.g);
(6) 0.2 g/kg storax group: ISO (sc) + 0.2 g/kg/d storax (i.g);
(7) 0.4 g/kg storax group: ISO (sc) + 0.4 g/kg/d storax (i.g).

**Figure 10:**
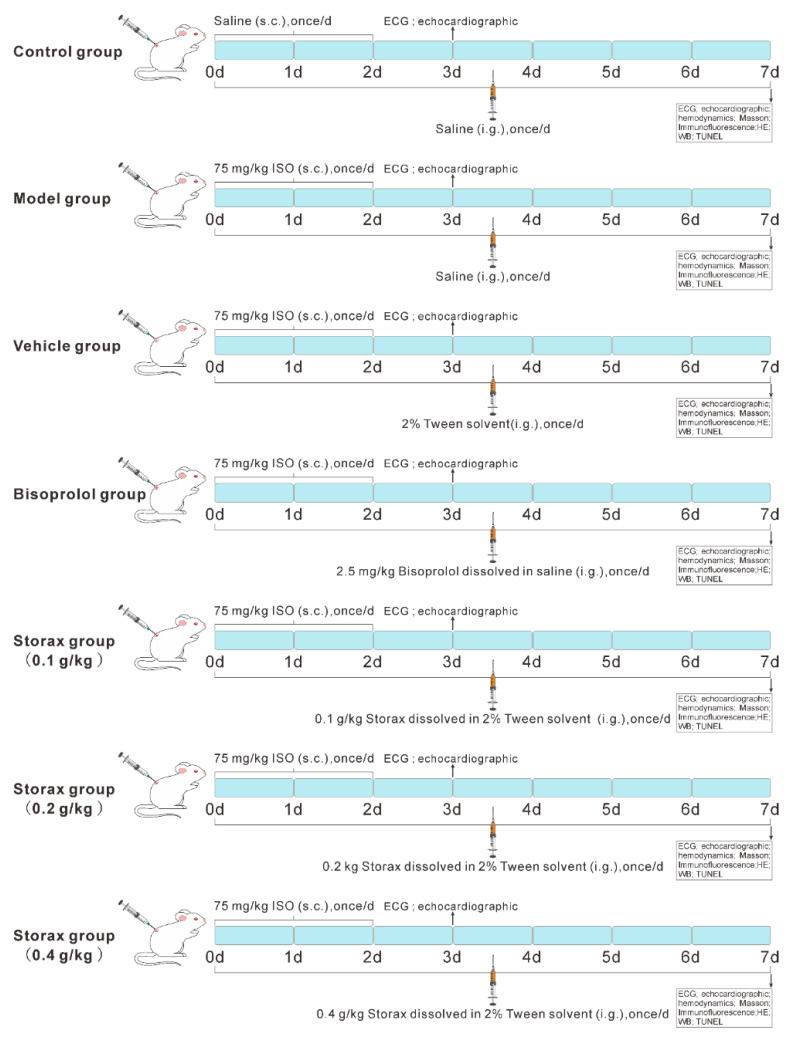
Experimental design for cardioprotective study of storax in rats.

Rats received continuous intragastric administration for 7 d. Experimental design for cardioprotective study of storax in rats is shown in Figure 10.

### 4.4. Cardiac Electrophysiology and Cardiac-Function-Related Information Collection

#### 4.4.1. Dynamic Monitoring of Electrocardiogram

On day 3 and day 7, the rats were anesthetized with isoflurane 30 min after administration. After the rats entered anesthesia, the breathing was stable and the pain disappeared; the rats were fixed on the test bench in the supine position. Use needle electrodes to insert subcutaneously into the limbs of rats (be careful not to insert them into muscles). Connect to BL-420 four-channel biosignal analysis system (Chengdu Taimeng Software Co., Ltd.). Use limb II lead to monitor the electrocardiogram of rats. Press the right upper limb: white, right lower limb: black, left lower limb: red. Record the electrocardiogram 15 min after the rat has stabilized. The changes in HR, ST-segment amplitude and Q-wave amplitude of rat electrocardiogram were analyzed, and pathological Q-wave and/or ST-segment displacement greater than 0.2 mV in the model group was used as the mark of successful AMI modeling [53].

#### 4.4.2. Dynamic Monitoring of Echocardiography

On day 3 and day 7, echocardiography was performed immediately after the ECG was detected. The chest hair of the rats was removed one day before the test. After isoflurane anesthesia, transthoracic echocardiograms were acquired with a Vevo 3100 small animal ultrasound system (FUJIFILM VisualSonics, Inc., Toronto, Ontario, Canada). The MX 250 ultrasonic probe was selected with a frequency of 21 MHz. The parasternal left ventricular short axis was taken, and the measured indexes included LVIDs, LVESV, EF and FS. On day 7, the long-axis section of the left ventricle was taken, and Doppler mode was used to detect the E peak of the mitral valve blood flow velocity in the early diastole and the A peak of the mitral valve blood flow velocity in the late diastole, and the E/A value was calculated. Three consecutive cardiac cycles were detected and the average value was taken to reflect the morphological structure and diastolic and systolic function of the left heart.

#### 4.4.3. Measurement of Hemodynamics

After the second echocardiographic examination, the rats were anesthetized by intraperitoneal injection of 20% urethane (0.6 mL/100 g), supine immobilized and depilated on the neck. A longitudinal incision was made on the lateral side of the right neck. The muscle was bluntly dissected layer by layer until the common carotid artery was exposed and carefully dissected, and the common carotid artery was isolated and placed under it with a knife handle. Thread a thread under the artery, ligate the distal end and clamp the proximal end with an artery clip. Cut a small incision with arterial scissors between the arterial clip and the ligation line near the ligation line and insert a PE 50 plastic catheter filled with 2% heparin saline into the common carotid artery by gentle rotation. Release the arterial clip and advance the catheter by 0.5 to 1 cm to record the peripheral blood pressure. After the recording, the catheter is gently rotated according to the pressure waveform and sent to the left ventricle. When the pressure waveform suddenly becomes steep, it indicates that it enters the left ventricle. At this time, the arterial clip clamped and fixed the catheter and recorded and analyzed MAP, SBP, DBP, LVSP, LVDP, LVEDP, LVP, ±dp/dt max, Vmax and T. After 15 min of stable recording, the proximal end of the catheter was removed with an arterial clip.

### 4.5. Measurement of Myocardial Injury Biomarkers

Blood samples of rats were collected from the abdominal aorta and rested for 1 h. The serum was obtained by centrifugation at 3500× *g* for 15 min. The levels of LDH, AST, CK-MB and α-HBDH in rat serum were measured using a fully automated blood biochemistry instrument (BS-240VET, mindray, Shenzhen, China).

### 4.6. Myocardial Tissue and Myocardial Fiber Morphology Observation

#### 4.6.1. Myocardial Histopathological Examination

After euthanasia of the rats, the hearts of the rats were cut along the root of the aorta. The residual blood was washed several times in ice-cold PBS and blotted dry with filter paper. Myocardial tissue was cut along the longitudinal axis of the left ventricle from the apex to the base of the heart and fixed in 10% formalin solution, paraffin-embedded and sectioned. The myocardial tissue was stained with hematoxylin–eosin (HE) or Masson stain to assess the histopathological morphology and the degree of fibrosis, respectively. Further, each group of stained sections was imaged and scanned with a NanoZOOMER S60 digital section scanner (Hamamatsu, Shizuoka, Japan). Myocardial morphology was observed at objective effects of 10× as well as 40×, respectively. Five different fields of Masson staining were selected for each section, and fibrosis area was detected by Image Lab 3.0 software.

#### 4.6.2. Immunofluorescence Detection

Paraffin sections were sequentially placed in xylene I for 15 min, xylene Ⅱ for 15 min, anhydrous ethanol I for 5 min, anhydrous ethanol Ⅱ for 5 min, 85% alcohol for 5 min, 75% alcohol for 5 min and distilled water to elute the wax. Antigen repair was subsequently performed using citric acid buffer. Sections were slightly shaken dry and circles were drawn around the tissue with a histochemical pen. Autofluorescence quencher was added to the circles for 5 min. Rinsed in running water for 10 min. BSA was added dropwise to close the circles for 30 min. Primary antibodies prepared in a certain ratio were added dropwise to the sections (anti-collagen I rabbit pAb, GB11022-3, 1:500; anti-collagen Ⅲ rabbit pAb. GB111629, 1:500; Servicebio, Wuhan, China). The sections were washed three times for 5 min each time by shaking in PBS on a decolorizing shaker. The sections were slightly shaken and dried, and the tissue was covered with secondary antibodies of the corresponding species in the circle dropwise and incubated for 50 min at room temperature, protected from light. The nuclei were re-stained with DAPI, and the sections were sealed with anti-fluorescence quenching sealer. The stained sections were observed and images were acquired on a 3Dhistech Kft Pannoramic Scan Ⅱ digital slide scanning system (Budapest, Hungary). Five field of view images were selected from each section, and the images were subsequently imported into Image J software for analysis.

#### 4.6.3. TUNEL Staining

Paraffin sections were dewaxed to water. The sections were incubated with proteinase K working solution for 22 min at 37 °C, washed 3 times with PBS and incubated with 0.1% triton for 20 min. After PBS washing, the sections were incubated dropwise with buffer for 10 min at room temperature. Appropriate amounts of TDTase, dUTP and buffer were mixed in the ratio of 1:5:50 according to the instructions of the TUNEL staining kit. The sections were incubated at 37 °C for 2 h. After washing with PBS, DAPI staining solution was added dropwise, and the sections were incubated for 10 min at room temperature, protected from light and sealed with anti-fluorescence quenching sealer. The stained sections were observed and images were acquired on a 3Dhistech Kft Pannoramic Scan II digital slide scanning system. Five field of view images were selected for each section, and the images were subsequently imported into Image Lab 3.0 software for analysis.

### 4.7. WB Analysis

Protein samples were extracted from each group of rat myocardial tissues using RIPA lysis solution containing 1% phosphatase and protease inhibitors. After homogenization in the homogenizer, the samples were lysed on ice for 15 min followed by ultrasonic fragmentation using a SCIENTZ-IID touch ultrasonic cell crusher (Ningbo Xinzhi Biotechnology Co., Ltd., Ningbo, China). At 40% output power on ice, sonication was performed 3 times for 10 s each with 15 s interval and centrifuged. The supernatant was taken to determine the protein concentration using the BCA kit and then adjusted to 30 ug for protein loading. Separated using 10–15% SDS-PAGE and transferred to PVDF membrane at 300 mA for 30 min. These membranes were closed with 5–10% skimmed milk powder (TBST preparation) at room temperature for 2 h. Incubated with primary antibody at 4 °C overnight, respectively (as shown in Table 1), and then with HRP secondary antibody at room temperature for 1.5 h. β-tublin, GAPDH can be used as internal reference. The strips were dropwise added with UltraSignal ultrasensitive ECL chemiluminescence substrate and then placed on GelView 6000Plus intelligent chemiluminescence image workstation (Guangzhou Boluteng Biotechnology Co., Ltd., Guangzhou, China) for development. Subsequently, semi-quantitative analysis was performed using Image Lab 3.0 software.

### 4.8. Statistical Analysis

All data are presented as mean ± SD of at least three independent experiments. Differences between groups were analyzed using one-way ANOVA, and LSD test or Dunnett *t*-test was used for pairwise comparisons. Brown–Forsythe and Welch ANOVA tests were used when variance was not equal, and *p* < 0.05 indicates statistical significance. Statistical analysis and graphing were performed using SPSS 25 or GraphPad Prism 8 software. 

## 5. Conclusions

In a nutshell, the current study indicated that storax could improve cardiac function and reduce interstitial collagen fiber deposition in AMI rats. It was initially demonstrated that the mechanism of storax against myocardial fibrosis and cardiac dysfunction was achieved through suppression of AT1R–Ankrd1–P53-pathway-mediated apoptosis. It expanded the application space of storax and provided an experimental basis for its clinical treatment. Of course, the mechanisms of myocardial fibrosis are complex, the pathways of apoptosis are diverse and other mechanistic pathways remain to be evaluated in future studies.

## Figures and Tables

**Table 1:** Primary antibody used for Western blot in this study.

Protein	Primary Antibody	Concentration
AT1R	Anti-AT1R, GB112004, Servicebio	1:1000
Ankrd1	Anti-CARP, AF0677, Affinity	1:1000
P53	Anti-P53, 2524S, CST	1:1000
P-p53 (Ser15)	Anti-P-p53 (Ser15), AF3075, Affinity	1:1000
Mdm2	Anti-Mdm2, AF0208, Affinity	1:1000
Bax	Anti-Bax, T40051, Abmart	1:1000
Bcl-2	Anti-Bcl-2, T40056, Abmart	1:1000
Cleaved Caspase-3	Cleaved Caspase-3, AF7022, Affinity	1:1000
GAPDH	Anti-GAPDH, AF7021, Affinity	1:5000
β-tubulin	Anti-β-tubulin, AF7011, Affinity	1:5000

## Data Availability

Not applicable.
